# Use of Hydrocortisone Based on Plasma Biomarkers in Patients with Septic Shock: Another One Bites the Dust?

**DOI:** 10.1164/rccm.202005-1984ED

**Published:** 2020-09-01

**Authors:** Morten Hylander Møller, Manu Shankar-Hari, Waleed Alhazzani

**Affiliations:** ^1^ Department of Intensive Care 4131; ^2^ Collaboration for Research in Intensive Care Copenhagen University Hospital Rigshospitalet Copenhagen, Denmark; ^3^ School of Immunology and Microbial Science Kings College London London, United Kingdom; ^4^ Guy’s and St. Thomas’ NHS Foundation Trust St. Thomas’ Hospital London, United Kingdom; ^5^ Department of Health Research Methods, Evidence, and Impact McMaster University Hamilton, Ontario, Canada and; ^6^ Department of Medicine McMaster University Hamilton, Ontario, Canada

In 2018, the ADRENAL (Adjunctive Glucocorticoid Therapy in Patients with Septic Shock) and the APROCCHSS (Hydrocortisone Plus Fludrocortisone for Adults with Septic Shock) trials were published (1, 2). Both trials compared the use of hydrocortisone versus placebo in adults with septic shock and found that hydrocortisone reduced time on mechanical ventilation, time to resolution of shock, and time in the ICU. Although the APROCCHSS trial showed a reduction in mortality with hydrocortisone use, this was not confirmed in the ADRENAL trial. Data from APROCCHSS and ADRENAL were subsequently included in updated systematic reviews and meta-analyses, which confirmed the findings from the two large trials (3, 4). Based on this, an international clinical practice guideline proposed a weak recommendation in favor of corticosteroids in patients with sepsis, including septic shock (5). Although most clinicians—based on the results of the trials and systematic reviews and on the proposed weak recommendation in the clinical practice guideline—may consider using hydrocortisone in patients with septic shock, especially in those with refractory shock, there is more uncertainty about the value of treatment with hydrocortisone based on plasma biomarker levels.

In this issue of the *Journal*, Cohen and colleagues (pp. 700–707) report the results of a cohort study nested within the ADRENAL trial (6). The authors assessed whether prerandomization baseline levels of the plasma biomarkers cortisol, aldosterone, and ascorbic acid modified the effect of hydrocortisone among 529 patients with septic shock from 22 ICUs in Australia and New Zealand. The authors observed no statistically significant interaction between plasma concentrations of the biomarkers and adjunctive hydrocortisone on 90-day mortality (primary outcome measure), shock reversal, or any other of the clinically relevant secondary outcomes, suggesting that the treatment effect of hydrocortisone is not dependent on the baseline plasma concentrations of cortisol, aldosterone, or ascorbic acid. There was an indication of interaction for free and total plasma concentrations of cortisol for some secondary outcomes and analyses; however, those analyses were not adjusted for multiple testing and therefore should be interpreted with caution, as acknowledged by the authors.

There are many strengths of the study. Embedding the study in a high-quality randomized clinical trial ensured prospective valid data collection with few missing data, including few patients lost to follow-up. The internal and external validity is high, and the results are applicable to other ICUs with a similar case mix. The comprehensive and prespecified statistical analysis plan, including the heterogeneity of treatment effect analysis, increases the transparency and robustness of the results. The sample size was large with resulting precise estimates. The use of reliable techniques to measure the biomarkers increases the validity of the measurements.

Despite the many strengths, the study holds some limitations. Sampling 14% (529/3,800) of the ADRENAL trial participants and limiting the sampling to baseline-only measurements may have introduced bias. Although robust, prespecified, and comprehensive, the statistical analyses were not adjusted for multiplicity, which may have resulted in false positives (type 1 error). Finally, patients included in the ADRENAL trial did not receive fludrocortisone along with hydrocortisone, therefore limiting the applicability to clinical settings with similar practice.

The clinical implications from this important, well-planned, and reported ADRENAL substudy are multiple. First and foremost, the finding that patients with lower or higher concentrations of baseline total or free cortisol concentrations did not receive any greater or lesser clinical effect from hydrocortisone treatment suggests that initial plasma cortisol is not a useful biomarker to identify patients who may benefit from this treatment. Owing to the known undesirable effects of hydrocortisone (3, 4, 7), use of hydrocortisone outside the pragmatic clinical criteria used in the APROCCHSS or in the ADRENAL trials is discouraged. Second, there is no indication of any clinically relevant association between baseline aldosterone concentrations and patient-important outcomes, which may suggest that mineralocorticoid deficiency plays a limited role in the pathophysiology in septic shock. Finally, there is evidence that low levels of ascorbic acid (hypovitaminosis C) plays a limited role in patients with septic shock, which indirectly questions the proposed combination therapy with ascorbic acid, hydrocortisone, and thiamine as an effective treatment strategy in septic shock (8). This is further supported by the recently published CITRIS-ALI (Effect of Vitamin C Infusion on Organ Failure and Biomarkers of Inflammation and Vascular Injury in Patients with Sepsis and Severe Acute Respiratory Failure) and VITAMINS (Effect of Vitamin C, Hydrocortisone, and Thiamine versus Hydrocortisone Alone on Time Alive and Free of Vasopressor Support among Patients with Septic Shock) trials, which could not confirm any benefit from use of this combination therapy (9, 10).

An important inference is that baseline levels of cortisol, aldosterone, and ascorbic acid do not seem to influence the treatment response of hydrocortisone, which may question the proposed treatment effect heterogeneity in patients with sepsis (11). However, differences in treatment effect to corticosteroids could be influenced by sepsis subtypes (12). These results, along with the fact that corticosteroids are a commonly used low-cost intervention, are an argument for finding patient characteristics that are associated with increased risk of harm with this intervention, rather than focusing on treatment response and efficacy.

In summary, there is no suggestion of heterogeneity in the effect of adjunctive hydrocortisone on mortality, shock resolution, or other clinical outcomes based on cortisol, aldosterone, or ascorbic acid concentrations in patients with septic shock. Consequently, there is no value in measuring these biomarkers at baseline to determine the treatment response of hydrocortisone in patients with septic shock. At best, it makes no difference and is a waste of resources; at worst, it is associated with undesirable effects, including hypernatremia, hyperglycemia, and neuromuscular weakness (5).

## Supplementary Material

Supplements

Author disclosures

## References

[1] Annane D Renault A Brun-Buisson C Megarbane B Quenot JP Siami S *et al* CRICS-TRIGGERSEP Network Hydrocortisone plus fludrocortisone for adults with septic shock *N Engl J Med* 2018 378 809 818 29490185 10.1056/NEJMoa1705716

[2] Venkatesh B Finfer S Cohen J Rajbhandari D Arabi Y Bellomo R *et al* ADRENAL Trial Investigators and the Australian–New Zealand Intensive Care Society Clinical Trials Group Adjunctive glucocorticoid therapy in patients with septic shock *N Engl J Med* 2018 378 797 808 29347874 10.1056/NEJMoa1705835

[3] Rygård SL Butler E Granholm A Møller MH Cohen J Finfer S *et al* Low-dose corticosteroids for adult patients with septic shock: a systematic review with meta-analysis and trial sequential analysis *Intensive Care Med* 2018 44 1003 1016 29761216 10.1007/s00134-018-5197-6

[4] Rochwerg B Oczkowski SJ Siemieniuk RAC Agoritsas T Belley-Cote E D’Aragon F *et al* Corticosteroids in sepsis: an updated systematic review and meta-analysis *Crit Care Med* 2018 46 1411 1420 29979221 10.1097/CCM.0000000000003262

[5] Lamontagne F Rochwerg B Lytvyn L Guyatt GH Møller MH Annane D *et al* Corticosteroid therapy for sepsis: a clinical practice guideline *BMJ* 2018 362 k3284 30097460 10.1136/bmj.k3284 PMC6083439

[6] Cohen J Bellomo R Billot L Burrell LM Evans DM Finfer S *et al* Plasma cortisol, aldosterone, and ascorbic acid concentrations in patients with septic shock do not predict treatment effect of hydrocortisone on mortality: a nested cohort study *Am J Respir Crit Care Med* 2020 202 700 707 32396775 10.1164/rccm.202002-0281OC

[7] Butler E Møller MH Cook O Granholm A Penketh J Rygård SL *et al* The effect of systemic corticosteroids on the incidence of gastrointestinal bleeding in critically ill adults: a systematic review with meta-analysis *Intensive Care Med* 2019 45 1540 1549 31501997 10.1007/s00134-019-05754-3

[8] Marik PE Khangoora V Rivera R Hooper MH Catravas J Hydrocortisone, vitamin C, and thiamine for the treatment of severe sepsis and septic shock: a retrospective before-after study *Chest* 2017 151 1229 1238 27940189 10.1016/j.chest.2016.11.036

[9] Fujii T Luethi N Young PJ Frei DR Eastwood GM French CJ *et al* VITAMINS Trial Investigators Effect of vitamin C, hydrocortisone, and thiamine vs hydrocortisone alone on time alive and free of vasopressor support among patients with septic shock: the VITAMINS randomized clinical trial *JAMA* 2020 323 423 431 31950979 10.1001/jama.2019.22176 PMC7029761

[10] Fowler AA III Truwit JD Hite RD Morris PE DeWilde C Priday A *et al* Effect of vitamin C infusion on organ failure and biomarkers of inflammation and vascular injury in patients with sepsis and severe acute respiratory failure: the CITRIS-ALI randomized clinical trial *JAMA* 2019 322 1261 1270 31573637 10.1001/jama.2019.11825 PMC6777268

[11] Santhakumaran S Gordon A Prevost AT O’Kane C McAuley DF Shankar-Hari M Heterogeneity of treatment effect by baseline risk of mortality in critically ill patients: re-analysis of three recent sepsis and ARDS randomised controlled trials *Crit Care* 2019 23 156 31053084 10.1186/s13054-019-2446-1 PMC6500045

[12] Antcliffe DB Burnham KL Al-Beidh F Santhakumaran S Brett SJ Hinds CJ *et al* Transcriptomic signatures in sepsis and a differential response to steroids: from the VANISH randomized trial *Am J Respir Crit Care Med* 2019 199 980 986 30365341 10.1164/rccm.201807-1419OC PMC6467319

