# Supplementary material for: Use of Hydrocortisone Based on Plasma Biomarkers in Patients with Septic Shock: Another One Bites the Dust?
Source: Am J Respir Crit Care Med. 2020 Sep 1;202(5):644–6. doi: 10.1164/rccm.202005-1984ED (PMC7462389; doi:10.1164/rccm.202005-1984ED)
Supplement: Supplements [file rccm.202005-1984ED.html]

Use of Hydrocortisone Based on Plasma Biomarkers in Patients with Septic Shock: Another One Bites the Dust? | American Journal of Respiratory and Critical Care Medicine

- disclosures.pdf (164 KB)
